# Draft Genome Sequence of the Gram-Positive Neutrophilic Iron-Precipitating *Kineosporia* sp. Strain A_224

**DOI:** 10.1128/genomeA.00763-17

**Published:** 2017-08-10

**Authors:** Burga Braun, Sven Künzel, Ulrich Szewzyk

**Affiliations:** a Technische Universität Berlin, Berlin, Germany; b Max Planck Institute for Evolutionary Biology, Plön, Germany

## Abstract

We report here the draft genome sequence of the neutrophilic iron-precipitating *Kineosporia* sp. strain A_224. Analysis of the predicted genes may improve our knowledge of its role in ochrous formations in natural and technical water systems. This is the first public genome sequence of a *Kineosporia aurantiaca* strain.

## GENOME ANNOUNCEMENT

A novel iron-depositing actinobacterial strain of the family *Kineosporiaceae* was isolated from the floodplain area in the Lower Oder Valley National Park, Germany. Based on 16S rRNA gene sequence analysis, the family *Kineosporiaceae* (1) includes the five genera *Angustibacter* (2), *Kineococcus* (3), *Kineosporia* (4), *Pseudokineococcus* (5), and *Quadrisphaera*. The genus *Kineosporia* contains seven species, *K. aurantiaca* (4), *K. babensis* (6), *K. mesophila* (7), *K. mikuniensis*, *K. rhamnosa*, *K. rhizophila*, and *K. succinea* (8). Iron-oxidation ability in the *Actinobacteria* family has been reported for only some acidic actinobacteria, such as members of the *Rubrobacteridae* subclass (9), the *Acidimicrobidae* subclass (10, 11), and the TM3 group of uncultured actinobacteria (12).

*Kineosporia* sp. strain A_224 was obtained from an iron-depositing biofilm of the floodplain area in the Lower Oder Valley National Park, Germany. The strain was isolated by spreading the suspended biofilm on ATA medium (13). Dark, brown-colored colonies that formed on the agar surface were selected after 2 weeks of incubation at room temperature. Iron-deposition ability of the strain was confirmed according to Schmidt et al. (14). Total genomic DNA was extracted using the GeneMATRIX soil DNA purification kit (Roboklon, Berlin, Germany). The paired-end library was prepared by following the Illumina Nextera XT DNA library prep kit protocol. Genome sequencing was done on an Illumina NextSeq 500 sequencer using the NextSeq mid-output kit version 2 with 300-cycle chemistry, generating 25,537,780 raw reads. Demultiplexing was done with bcl2fastq version 2.18.0.12. Quality filtering of the raw reads was performed using Trimmomatic version 0.36 (1). Reads were checked for ambiguous base calls and low complexity with the DUST algorithm (2) and were filtered accordingly with an R script in Microsoft R Open version 3.3.2 (3), followed by preassembly with SPAdes version 3.10.0 (4) using default *k*-mer lengths up to 99 bp. Scaffolds of this preassembly ≥500 bp were subject to extension and second-round scaffolding with SSPACE standard version 3.0 (5). Scaffolds ≥2,500 bp were assigned to genome bins by MetaBAT version 0.32.4 (8), and functional annotation of draft genomes was performed with Prokka version 1.12b (9). The shortest scaffold was 2,712 bp, and the longest scaffold was 335,770 bp. The total size of the draft genome was 7,196,381 bp with a GC content of 74%. Annotation resulted in 242 contigs, including 6,546 coding sequences for 6,626 genes, 1 CRISPR repeat unit, 3 rRNAs (5S, 16S, 23S), 63 tRNAs, 1 tmRNA, 13 miscellaneous RNAs, and 530 signal peptide coding sequences. Using the 16S rRNA gene, BLASTn searches (15) revealed a 96% gene sequence similarity to *K. aurantiaca* strain 14067 (NR_041718), and EzBioCloud (16) searches revealed a 96.5% similarity to *K. mesophila* strain YIM 65293 (FJ214362).

### Accession number(s).

This genome shotgun project has been deposited in GenBank under the accession number MWLN00000000. The version described in this paper is the first version, MWLN01000000.

## References

[1] Zhi XY, Li WJ, Stackebrandt E 2009 An update of the structure and 16S rRNA gene sequence-based definition of higher ranks of the class *Actinobacteria*, with the proposal of two new suborders and four new families and emended descriptions of the existing higher taxa. Int J Syst Evol Microbiol 59:589–608. doi:10.1099/ijs.0.65780-0. 19244447

[2] Kim SJ, Jang YH, Hamada M, Tamura T, Ahn JH, Weon HY, Suzuki Ki, Kwon SW 2013 *Angustibacter* *aerolatus* sp. nov., isolated from air. Int J Syst Evol Microbiol 63:610–615. doi:10.1099/ijs.0.042218-0. 22544785

[3] Yokota A, Tamura T, Nishii T, Hasegawa T 1993 *Kineococcus* *aurantiacus* gen. nov., sp. nov., a new aerobic, Gram-positive, motile coccus with meso-diaminopimelic acid and arabinogalactan in the cell wall. Int J Syst Bacteriol 43:52–57. doi:10.1099/00207713-43-1-52.

[4] Pagani H, Parenti F 1978 Kineosporia, a new genus of the order *Actinomycetales*. Int J Syst Evol Microbiol 28:401–406.

[5] Jurado V, Laiz L, Ortiz-Martinez A, Groth I, Saiz-Jimenez C 2011 *Pseudokineococcus* *lusitanus* gen. nov., sp. nov., and reclassification of *Kineococcus* *marinus* Lee 2006 as *Pseudokineococcus* *marinus* comb. nov. Int J Syst Evol Microbiol 61:2515–2519. doi:10.1099/ijs.0.026195-0. 21112988

[6] Sakiyama Y, Thao NKN, Giang NM, Miyadoh S, Hop DV, Ando K 2009 *Kineosporia* *babensis* sp. nov., isolated from plant litter in Vietnam. Int J Syst Evol Microbiol 59:550–554. doi:10.1099/ijs.0.002907-0. 19244439

[7] Li J, Zhao G-Z, Huang H-Y, Qin S, Zhu W-Y, Xu L-H, Li W-J 2009 *Kineosporia* *mesophila* sp. nov., isolated from surface-sterilized stems of *Tripterygium wilfordii*. Int J Syst Evol Microbiol 59:3150–3154. doi:10.1099/ijs.0.012021-0. 19643874

[8] Kudo T, Matsushima K, Itoh T, Sasaki J, Suzuki K-I 1998 Description of four new species of the genus *Kineosporia*: *Kineosporia* *succinea* sp. nov., *Kineosporia* *rhizophila* sp. nov., *Kineosporia* *mikuniensis* sp. nov. and *Kineosporia* *rhamnosa* sp. nov., isolated from plant samples, and amended description of the genus *Kineosporia*. Int J Syst Bacteriol 48:1245–1255. doi:10.1099/00207713-48-4-1245. 9828426

[9] Bryan CG, Johnson DB 2008 Dissimilatory ferrous iron oxidation at a low pH: a novel trait identified in the bacterial subclass *Rubrobacteridae*. FEMS Microbiol Lett 288:149–155. doi:10.1111/j.1574-6968.2008.01347.x. 18803673

[10] Johnson DB, Bacelar-Nicolau P, Okibe N, Thomas A, Hallberg KB 2009 *Ferrimicrobium* *acidiphilum* gen. nov., sp. nov. and *Ferrithrix* *thermotolerans* gen. nov., sp. nov.: heterotrophic, iron-oxidizing, extremely acidophilic actinobacteria. Int J Syst Evol Microbiol 59:1082–1089. doi:10.1099/ijs.0.65409-0. 19406797

[11] Norris PR, Davis-Belmar CS, Brown CF, Calvo-Bado LA 2011 Autotrophic, sulfur-oxidizing actinobacteria in acidic environments. Extremophiles 15:155–163. doi:10.1007/s00792-011-0358-3. 21308384

[12] Kanaparthi D, Pommerenke B, Casper P, Dumont MG 2013 Chemolithotrophic nitrate-dependent Fe(II)-oxidizing nature of actinobacterial subdivision lineage TM3. ISME J 7:1582–1594. doi:10.1038/ismej.2013.38. 23514778 PMC3721109

[13] Braun B, Szewzyk U 2016 Complete genome sequence of “Candidatus *Viadribacter manganicus*” isolated from a German floodplain. Area 4:3402. 10.1128/genomeA.00897-16 PMC5009972 27587815

[14] Schmidt B, Sánchez LA, Fretschner T, Kreps G, Ferrero MA, Siñeriz F, Szewzyk U 2014 Isolation of *Sphaerotilus–Leptothrix* strains from iron bacteria communities in Tierra del Fuego wetlands. FEMS Microbiol Ecol 90:454–466. doi:10.1111/1574-6941.12406. 25098830

[15] Altschul SF, Madden TL, Schaffer AA, Zhang J, Zhang Z, Miller W, Lipman DJ 1997 Gapped BLAST and PSI-BLAST: a new generation of protein database search programs. Nucleic Acids Res 25:3389–3402. doi:10.1093/nar/25.17.3389. 9254694 PMC146917

[16] Yoon S, Ha S, Kwon S, Lim J, Kim Y, Seo H, Chun J 2017 Introducing EzBioCloud: a taxonomically united database of 16S rRNA gene sequences and whole-genome assemblies. Int J Syst Evol Microbiol 67:1613–1617. 28005526 10.1099/ijsem.0.001755 PMC5563544

